# Biological and Proteomic Characteristics of an Immortalized Human Pancreatic Stellate Cell Line

**DOI:** 10.7150/ijms.36337

**Published:** 2020-01-01

**Authors:** Li Sun, Limei Qu, David R Brigstock, Hongyan Li, Yanyan Li, Runping Gao

**Affiliations:** 1 Department of Hepatic Biliary Pancreatic Medicine, First Hospital of Jilin University, Changchun, 130021, China; 2 Research Institute at Nationwide Children's Hospital, Columbus, 43205, United States

**Keywords:** human pancreatic stellate cell, immortalization, TGF-β1, pancreatic fibrosis, proteomics

## Abstract

Human pancreatic stellate cells (PSCs) play a critical role in fibrogenesis during chronic pancreatitis (CP). However, primary PSCs have a short lifespan *in vitro*, which seriously affects their use in various applications. We have established a stable immortalized human PSC line (HP-1) by RSV promoter/enhancer-driven SV40 T antigen expression in primary activated human PSCs. HP-1 cells express cytoskeleton proteins including glial fibrillary acidic protein (GFAP), α-smooth muscle actin (α-SMA), vimentin and desmin, and are typical of PSCs, which are high transfeciability and viable in 0.5% serum. The cells express receptors such as TGFβR2, PDGFR, TGF-β pseudoreceptor Bambi and PPRPγ that are commonly found in PSCs. HP-1 cells are similar to activated human PSCs in that they have enhanced expression of α-SMA, CTGF, Col1 and TIMP-2 mRNAs or proteins, as well as decreased expression of MMP-1/2 mRNAs or proteins in response to TGF-β1 stimulation. Comparative proteomics revealed 4,537 shared proteins between HP-1 cells and PSCs and no single protein in HP-1 cells versus PSCs. Statistical analysis reveals no significantly difference between HP-1 cells and PSCs in their expression of proteins associated with matrix and matrix remodeling. The similarity between HP-1 cell and PSC is further shown by the finding that only 9 proteins are differentially up-regulated > ± 2-fold in HP-1 cells and 13 proteins are up-regulated > ± 2-fold in PSCs and none of these proteins include ECM proteins, cytokines, growth factors or matrix remodeling regulatory proteins. Therefore, HP-1 cells can be used as an effective tool for the study of PSC-mediated pancreatic fibrosis.

## Introduction

Pancreatic fibrosis is a key pathological feature of chronic pancreatitis (CP) and pancreatic ductal adenoma in which the pancreatic stellate cells (PSCs) play an important role 1-4. In the normal pancreas, PSCs are located in periacinar and periductal regions and contain vitamin A in cytoplasmic lipid droplets. In response to pancreatic injury, they are transformed from a quiescent phenotype into myofibroblast-like cells, which lose vitamin A lipid droplets and express cytoskeleton proteins including alpha-smooth muscle actin(α-SMA), vimentin, desmin and glial fibrillary acidic protein (GFAP) 1, 3, 5. When cultured on uncoated plastic, PSCs undergo spontaneous activation that is similar to their reaction *in vivo* 6, 7. Activated PSCs can produce fibrogenic cytokine transforming growth factor β1 (TGF-β1) and mitogenic cytokine platelet-derived growth factor (PDGF), synthesize and secrete extracellular matrix (ECM) including type I collagen (Col1), and produce matrix metalloproteinases (MMP-1, MMP-2) and tissue inhibitors of matrix metaloproteinases (TIMPs). Therefore, PSCs play a key role in the pathogenesis of pancreatic fibrosis and deposition of large amounts of insoluble ECM 2, 5.

Primary cultures of human PSCs are terminally differentiated and cannot be maintained or passaged *in vitro* for long periods of time. To date, a few human PSC lines have been established by simian virus 40 (SV40)-driven SV40 large T antigen and human telomerase reverse transcriptase expression in primary PSCs 8, 9. However, these cell lines do not accurately represent the primary PSC phenotype due to their fast growth and continuous proliferation.

The aim of the present study was to develop a stable immortalized human PSC line by Rous sarcoma virus (RSV) promoter/enhancer-driven SV40 T antigen expression in primary activated PSCs, and to evaluate its potential as a valuable tool for the study of pancreatic fibrogenesis by analyzing and comparing the biological characteristics and proteomics between HP-1 cells and human PSCs.

## Materials and Methods

### Isolation and culture of human PSCs

Normal pancreatic tissue masses around a pancreatic cyst were obtained via routine pancreatic cystectomy at the First Hospital of Jilin University. The methods and experimental procedures in this study were performed in accordance with the Helsinki Declaration and approved by the ethics committee of the Hospital. The patient provided written informed consent to participate in this study. Human PSCs were isolated as previously described 6 but with a slightly modified. The detailed methodology about the isolation of human PSCs is described in Supplementary Material. Freshly isolated PSCs were cultured in DMEM supplemented with 25 mM Hepes buffer, 10% FBS, and 100 U/ml penicillin, 100 μg/ml streptomycin. The cells were maintained in a humidified 5% CO_2_ at 37ºC. Activated PSCs were split every 3 days at a ratio of 1:3 and used in the experiments at passage 3 to 6.

### Transfection and immortalization of human PSCs

Primary human PSCs at 24 days were plated in 6-well culture plates (1.25 × 10^5^/well). After incubation for 12 h, the medium was exchanged with fresh medium and the cells were transiently transfected for 24 h with FuGENE 6 Transfection Reagent (Promega, USA) and a plasmid that mediated the expression of SV40 T antigen by RSV promotor/enhancer. The cells were cultured for another 48 h and then placed in 10 cm diameter petri dish containing 2.5% FBS DMEM which were allowed to grow until 90% confluent. The cells were seeded at 3.0×10^5^ cells/dish and cultured in 2.5% FBS DMEM which subsequently underwent four passages every 7 days. For cell clone selection, the cells were seeded at 3.0×10^2^ cells/dish in the low serum medium and cultured up to the formation of immortal clones of cells. Six cell clones were selected and evaluated for their transformation phenotypes. A single cell clone, termed HP-1, that demonstrated a stable phenotype expressing desmin and closely resembling characteristics of activated PSC was selected and studied for over 60 generations. For the data reported here, HP-1 cells were used at passage 35 to 50. For comparison, non-immortalized human PSCs, obtained as described above, were harvested at passage 3 to 6 and are hereafter termed “PSCs”.

To determine the transfection efficiency of HP-1 cells, 1.5 × 10^5^ cells/well were placed in 4-well Lab-Tek® chamber slides. 2 μg of a plasmid expressing enhanced green fluorescence protein pEGFP-N1 was individually mixed with 4 μl FuGENE 6 or 10 μl X-treme GENE siRNA reagent in 100 μl DMEM for 15 min. The mixtures were respectively added to each well of the cells, which were cultured in 1% FBS DMEM for 24 h. The cells were stained with DAPI and visualized with an Olympus BX51 TRF fluorescent/light microscope (Olympus, Tokyo, Japan) for blue field and green fluorescence images. The proportion of GFP-positive cells among DAPI-positive cells was calculated from 10 randomly selected high-power field per specimen.

### Immunofluorescence staining

HP-1 cells were cultured in 4-well Lab-Tek® chamber slides. At the end of culture, slides were washed in PBS, and fixed in - 20ºC acetone for 30 min. Thereafter, slides were permeabilized with PBS containing 0.3% Triton-X100 for 30 min. The slide was incubated with mouse monoclonal antibodies to α-SMA (Boster, Wuhan, China) for 1 h at room temperature, followed by TRITC-labeled goat anti-mouse antibodies for 30 min. To identify the coexpression of the SV40 large T antigen and GFAP or vimentin and desmin, two slides were respectively incubated with primary antibodies to rabbit GFAP (ProteinTech, USA) and mouse SV40 Tag (Santa Cruz, USA) or rabbit desmin and mouse vimentin (Boster, Wuhan, China) at room temperature for 1 h, followed by goat anti-rabbit Alexa Fluor 555 (Life Technology, USA) and FITC labelled goat anti-mouse (Sigma, USA) secondary antibodies. Images were captured using Olympus BX51 TRF fluorescent/light microscope.

### Quantitative Real-Time PCR analysis

PSCs and HP-1 cells were placed 6-well culture plates and incubated in 1% FBS DMEM for 12 h in the absence or presence of TGF-β1 (5 ng/ml) or PDGF-BB (10 ng/ml). Total RNA was then extracted from the treated cells using Trizol reagent according to the manufacturer's instructions (TIANGEN, China). Levels of mRNA were determined by quantitative Real-Time PCR (qRT-PCR). Briefly, 1 µg RNA was reverse transcribed to cDNA using a RevertAid-First-Strand-cDNA-Synthesis-Kit. PCR analysis was performed using SYBR Green PCR Master Mix Kit (ThermoFisher Scientific, USA) with respective primer pairs on the Agilent Stratagene Mx3005p QPCR System. Data were normalized to β-actin, and fold change in target gene expression converted to Ct values using the Delta-Delta Ct method. qRT-PCR assays were repeated three times.

### Western blot analysis

PSCs or HP-1 cells were cultured for 24 h in the absence or presence of stimulators respectively. These cells were lysed using RIPA lysis buffer (Beyotime, China) containing protease inhibitor cocktail (Sigma, USA). Protein samples (15 μg each) from PSCs or HP-1 cells were separated on 10% SDS-PAGE gels, and transferred to nitrocellulose. After washing with Tris buffer saline containing 0.1% Tween 20 (TBS/T) and blocking with 2.5% non-fat milk, the membranes were separately incubated at 4℃ overnight with rabbit polyclonal anti-α-SMA, anti-CTGF, anti-Col1, anti-MMP-1, anti-MMP-2, anti-TIMP-2, anti-GAPDH antibodies (ProteinTech, USA). Blots were incubated for 1 h with HRP-linked goat anti-rabbit IgG (Beyotime), and washed extensively with TBS/T before detection using the ECL system (ThermoFisher Scientific, USA).

### Proteomics analysis

HP-1 cells or PSCs were placed in 10 cm dishes and cultured for 48 h in DMEM supplemented with 2.5% or 10% FBS respectively. Three samples of each cell type were lysed, digested with trypsinase, labeled by tandem mass tag (TMT), separated by EASY-nLC 1000 liquid chromatography system (Thermo Scientific, USA), and subjected to high-efficiency Q Exactive^TM^ Plus Orbitrap Fusion Mass Spectrometry (MS/MS) for protein detection and analysis (ThermoScientific, USA). All data generated were searched against Human UniProt database using the Mascot V2.3 search engine (Matrix Science, USA).

### Statistical analysis

The values reported represent mean ± SD of the measurements from at least 3 separate HP-1 cell and PSC cultures. Statistical analysis of the data was performed using SPSS 11.5 for Windows. The Student's t test was used for paired data. *P* value < 0.05 was considered significant. 1.5-fold change was used as the threshold of differential protein expression.

## Results

### Characteristics of human PSC cell line

**Figure 1:**
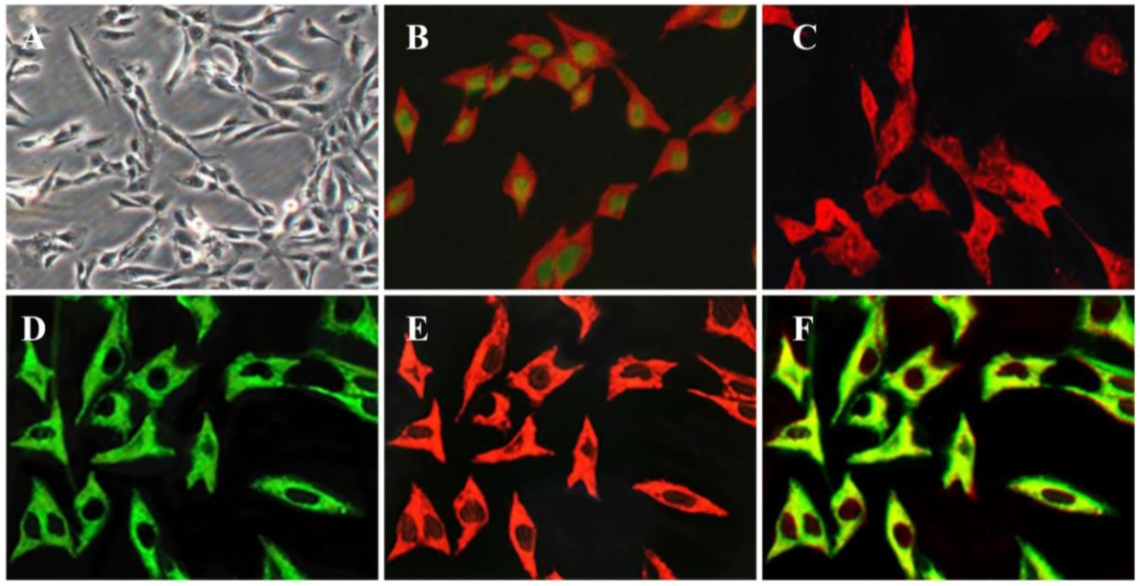
** Key characteristics of the immortalized HP-1 human PSC cell line.** Phase contrast microscopy in HP-1 cells after seeding for 12 h (**A**); Double-immunofluorescence labelling using antibodies for SV40 antigen and GFAP, green, localization of SV40 antigen, red, localization of GFAP (B); Immunofluorescence showing the presence of α-SMA (**C**); Double-immunofluorescence labelling for vimentin and desmin. Green, localization of vimentin (D), red, localization of desmin (E), yellow, colocalization of vimentin and desmin (F). (Original magnification × 200 in **A**; × 400 in **B-F**).

An immortalized human PSC cell line, HP-1, was successfully generated by transfection of primary human PSCs with RSV promoter/enhancer-driven SV40 T antigen. HP-1 cells exhibited a myofibroblast-like shape, and were morphologically very similar to primary, culture-activated PSCs. The cells were smaller in size, appearing less outstreached. (Fig. 1A). The cells expressed SV40 T antigen in their nuclei and also expressed cytoskeleton protein GFAP, α-SMA, vimentin, or desmin that is typical phenotypic characteristics of activated PSCs (Fig. 1B-E).

Since primary PSCs have shown desmin-positive or negative phenotypes 5, we determined whether the expression of desmin in HP-1 cells was changed during long time culture. As shown in Fig. 1D-F, the expression of desmin and vimentin was found to be colocalization and stable in all of the cells.

### Expression of receptors in human PSCs and HP-1 cells

**Figure 2:**
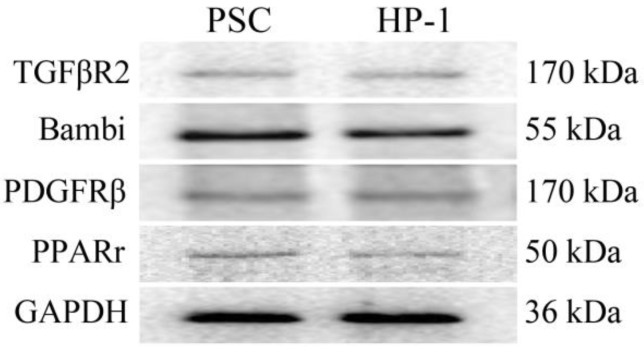
** Expression of receptors in PSCs and HP-1 cells.** Western blot showing similar expression of various receptors in PSCs and HP-1 cells.

In this study, PSCs and HP-1 cells showed high similarity in their respective expression of TGFβR2、Bambi、PDGFRβ or PPARγ (Fig. 2).

### TGF-β1 dependency of matrix and matrix remodeling proteins in human PSCs and HP-1 cells

**Figure 3:**
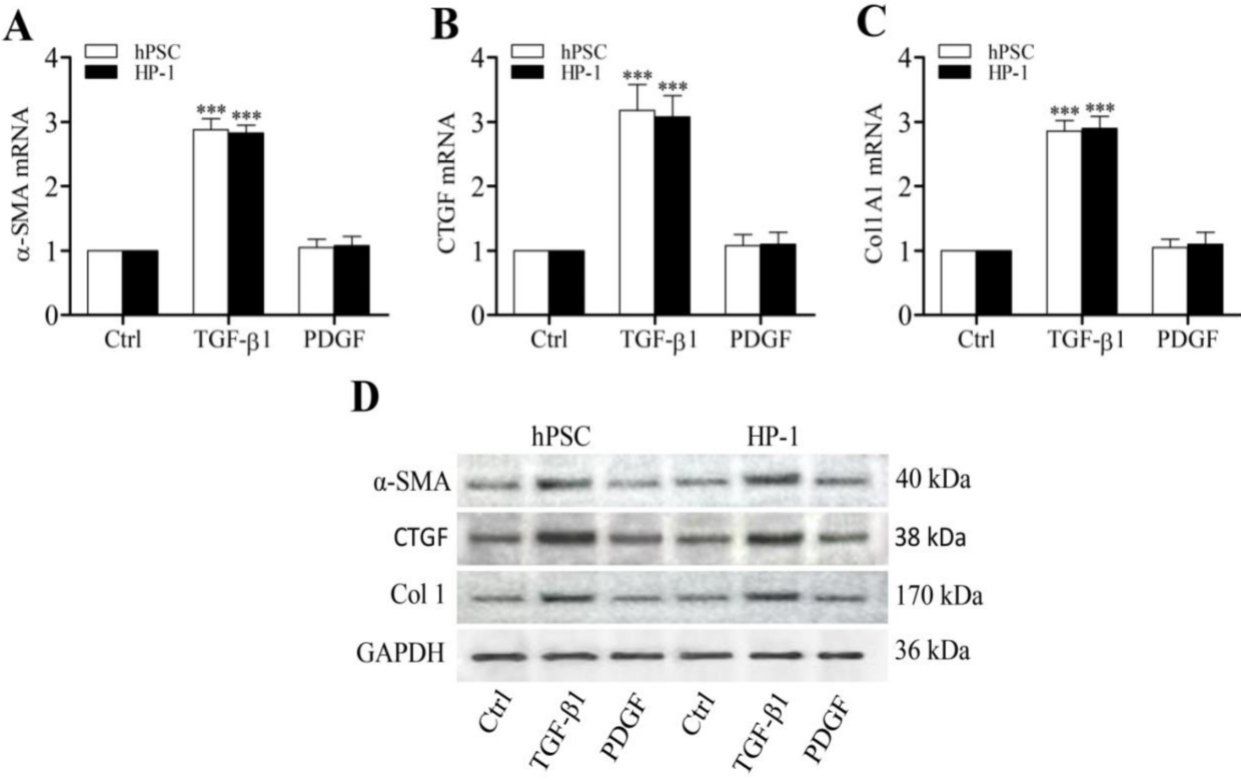
** Enhanced expression of α-SMA, CTGF and Col1 by TGF-β1**. RT-qPCR revealing the enhanced transcription of α-SMA (**A**), CTGF (**B**) and Col1A1 mRNAs (**C**) in PSCs and HP-1 cells by TGF-β1 but not PDGF; Western blot showing the increased expression of α-SMA, CTGF and Col1 proteins in PSCs and HP-2 cells consistent with their mRNA expression (**D**).

**Figure 4:**
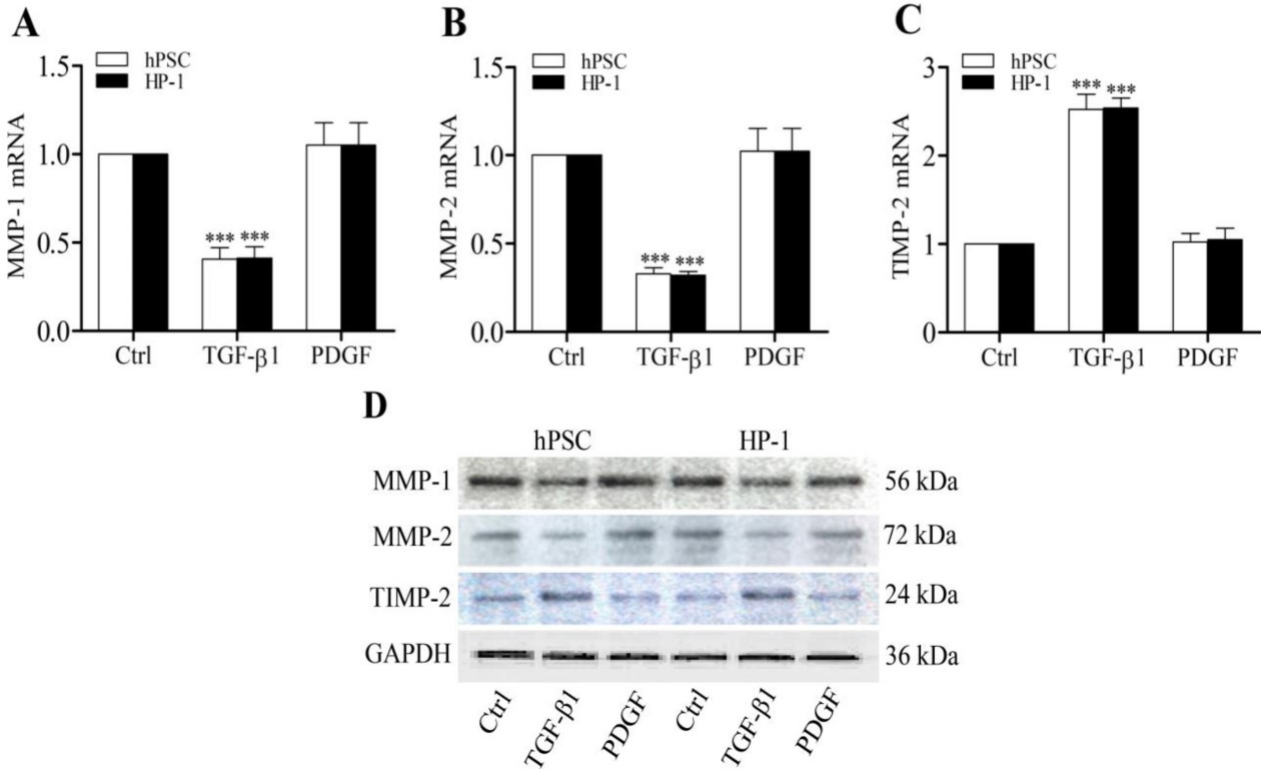
** TGF-β1 regulates MMP-1, MMP-2 and TIMP-2 production**. RT-qPCR showing decreased expression of MMP-1 (**A**) and MMP-2 (**B**), and increased expression of TIMP-2 mRNAs (**C**) in PSCs and HP-1 cells in response to TGF-β1 but not to PDGF; Western blot showing the expression of MMP-1, MMP-2 and TIMP-2 proteins in PSCs and HP-1 cells consistent with their mRNA expression (**D**).

Previously we demonstrated that CTGF functioned as a matricellular protein and acted as a downstream mediator of TGF-β-induced Col1 production in rat PSCs 5. In this study, TGF-β1-treated human PSCs and HP-1 cells showed higher levels of α-SMA, CTGF and Col1 mRNAs and proteins than control non-treated cells (Fig. 3A-D). Furthermore, TGF-β1-treated PSCs and HP-1 cells exhibited a decreased ability to produce MMP-1 or MMP-2 and an enhanced production of TIMP-2 mRNAs and proteins as compared to controls (Fig. 4A-D). In contrast, PDGF-treated PSCs and HP-1 cells demonstrated unchanged production α-SMA, CTGF, Col1A1, MMP-1, MMP-2 and TIMP-2. HP-1 cells showed high similarity with PSCs in the production of matrix and matrix remodeling protein (Fig. 3, Fig. 4).

### High transfection efficiency of HP-1 cells

**Figure 5:**
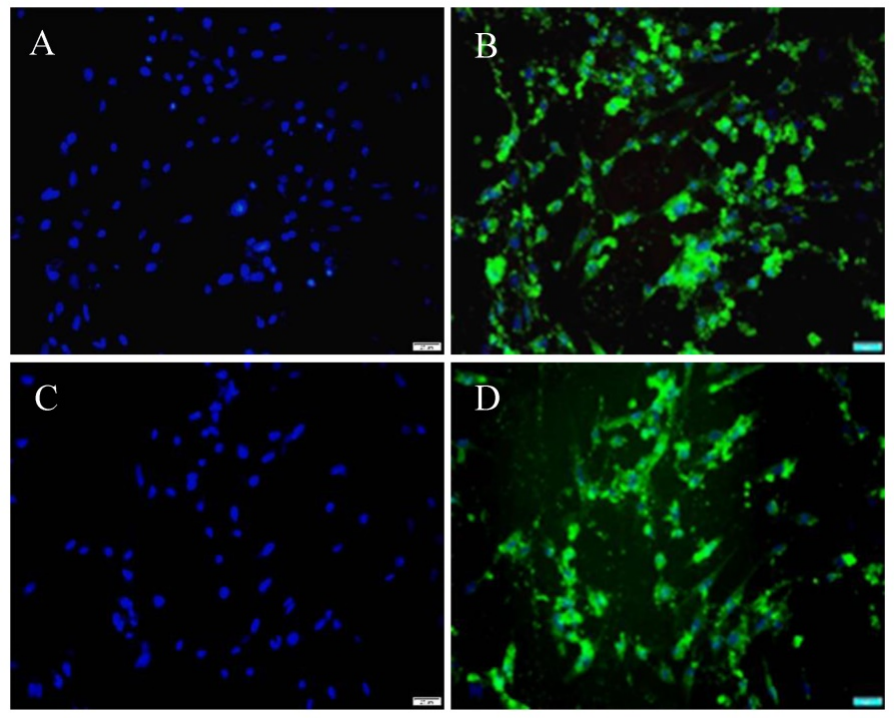
** HP-1 cells are highly transfectable.** HP-1 cells were plated in 4-well Lab-Tek Chamber slides for 12 h and then transfected with pEGFP-N1 and FuGENE 6 (**A, B**) or pEGFP-N1 and X-tremeGENE siRNA reagent (**C, D**). DAPI staining showing the area of the nucleus (Blue) (**A, C**); Green fluorescence of the same field of the cells (**B, D**) (Original magnification ×200 in **A-D**).

Historically, the low transfection efficiency (less than 30%) of cultured PSCs has been a considerable challenge for conducting mechanistic studies in these cells. In this study, cultured HP-1 cells were transfected with pEGFP-N1 using either FuGENE 6 or X-tremGENE siRNA reagent with the result that a high transfection efficiency was achieved with both lipofectamines (Fig. 5). The proportion of GFP-positive cells (Fig. 5B, D) among DAPI-positive cells (Fig. 5A, C) as calculated from 10 randomly selected high-power field per specimen was 61.8 ± 4.35% or and 88.65 ± 4.66% using, respectively, FuGENE 6 (Fig. 5A, B) or X-tremeGENE siRNA transfection agent (Fig. 5C, D).

### High similarity in proteomics between HP-1 cells and human PSCs

**Figure 6:**
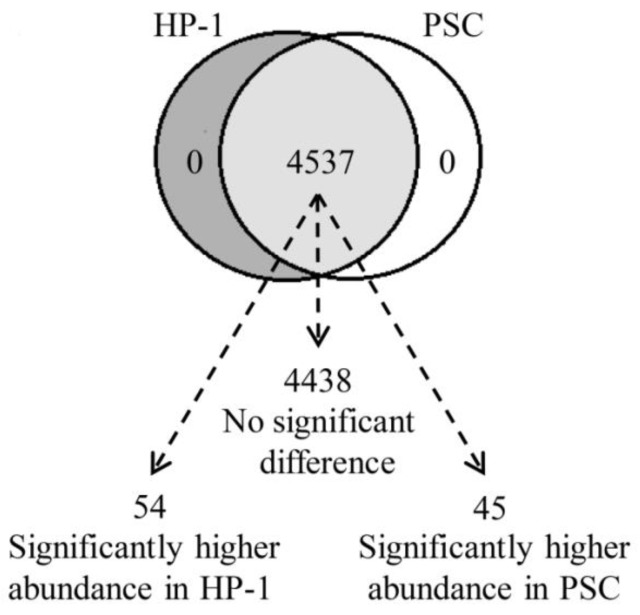
** The identified proteins in HP-1 cells is highly consistent with PSCs.** Venn diagram showing the number of identified and quantified proteins in HP-1 cells and PSCs. Of the 4537 quantified proteins, 4438 proteins are not significantly different between both cell types, 54 with significantly higher in the HP-1 cells and 45 significantly higher in the PSCs.

We used high-efficiency MS/MS-based quantitative proteomic strategy to compare the proteomics of HP-1 cells and PSCs. In total, we identified 4537 quantitative shared proteins in both cell types. Proteins that were expressed by HP-1 cells or PSCs alone were not identified. Based on p < 0.05 and fold change > ± 1.5, we found 54 up-regulated proteins in HP-1 cell, 45 up-regulated proteins in PSC, with no significant difference in expression of the remaining 4438 proteins (97.8% of the total quantifiable proteins in the two cell types) (Fig. 6).

The proteins identified above with differential expression of > ± 1.5-fold change were then sorted into six sub-ranges according to their actual level of differential expression. Most of these proteins showed a 1.5-2.0-fold change, and were demonstrated by 45 HP-1 cell proteins or 32 PSC proteins. Of the proteins that were differentially expressed at higher levels, 7 HP-1 proteins and 10 PSC proteins showed a 2.0-3.0-fold change while 2 HP-1 proteins and 3 PSC proteins showed a 3.0-4.0-fold change (Table 1).

**Figure 7:**
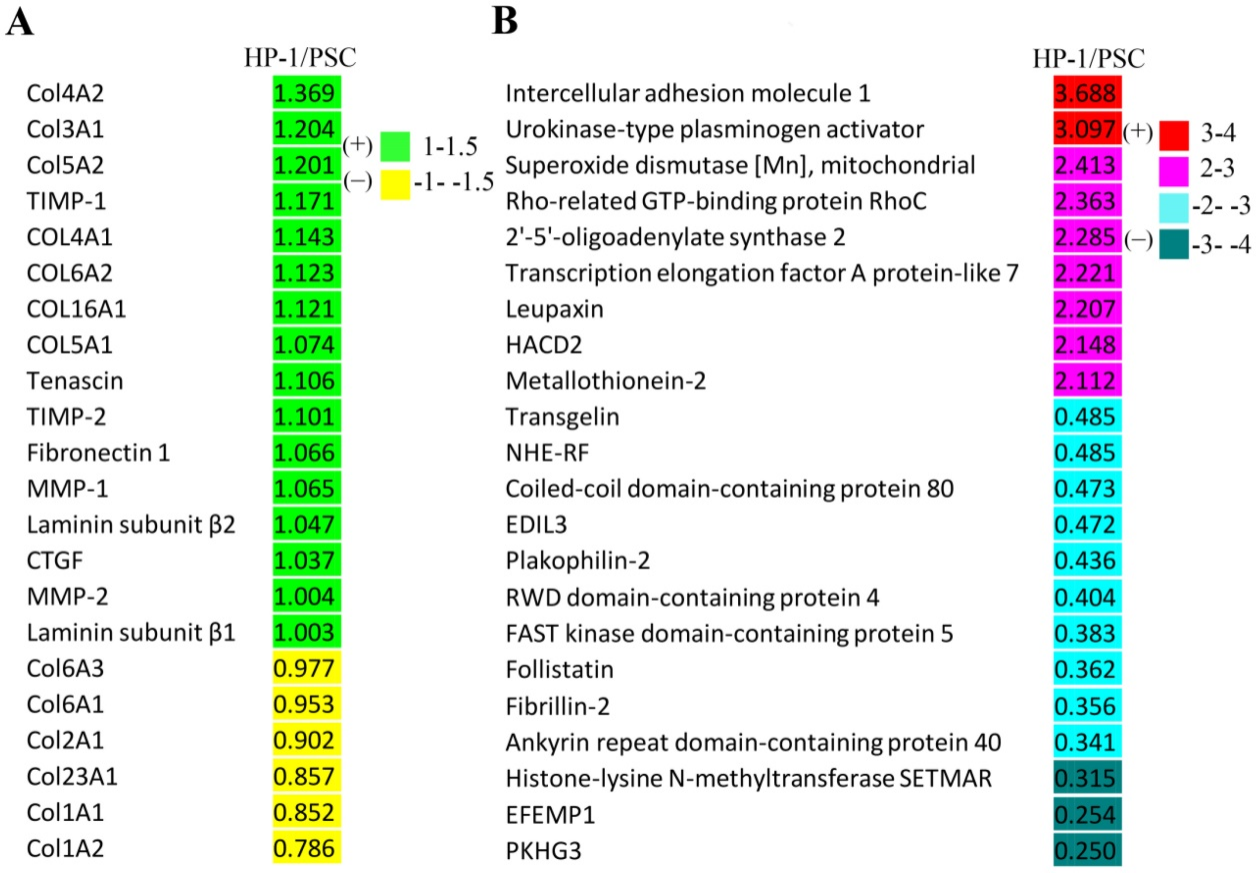
**Proteomic analysis of HP-1 cells versus PSCs. A:** Proteins including extracellular matrix and matrix metalloproteinases as well as their inhibitors identified were not significantly different (fold change **<** |**±**1.5|). **B**: Differentially-expressed proteins identified in proteomic analysis between HP-1 cells and PSCs (fold change **>** |**±**2|). HACD2: Very-long-chain (3R)-3-hydroxyacyl-CoA dehydratase 2; NHE-RF: Na (+)/H (+) exchange regulatory cofactor; EDIL3: EGF-like repeat and discoidin I -like domain -containing protein 3; EFEMP1: EGF-containing fibulin -like extracellular matrix protein 1; PKHG3: Pleckstrin homology domain -containing family G member 3.

As shown in Fig. 7A, the fold-changes of various ECM, matrix metalloproteinases and their inhibitors were less than |±1.5| as assessed by comparative proteomic analysis of HP-1 cell versus PSC. Additionally, the differentially-expressed proteins in HP-1 cells and PSCs with a fold change of more than |±2| are listed in Fig. 7B. The top two up-regulated proteins were intercellular adhesion molecule 1 and urokinase-type plasminogen activator, while the top three down-regulated proteins were histone-lysine N-methyltransferase, EGF-containing fibulin-like extracellular matrix protein 1, and pleckstrin homology domain- containing family G member 3. Since ECM, cytokine, growth factor and matrix remodeling regulatory proteins were not present in this list; the data suggest that the two cell types are closely aligned in their expression of these critical proteins.

## Discussion

Human PSCs play a key role in the fibrogenesis of chronic pancreatitis. However, studies on pancreatic stellate cells have not been carried out widely due to difficulty of maintaining primary PSCs in culture for a long time. So far a few human PSC lines have been established by simian virus 40 (SV40)-driven SV40 LT antigen and human telomerase expression in primary PSCs 8, 9. These cell lines do not accurately represent the phenotypic characteristics of primary PSC due to their fast growth and continuous proliferation. Since RSV promoter/enhancer contains stronger regulatory elements than SV40 for expression of genes in lymphoid cell lines 10, we have established a rat PSC line RP-2 cell by RSV-driven SV40 LT antigen expression 5. In this study we developed a stable immortalized human PSC line HP-1 cell by RSV promoter/enhancer-driven SV40 T antigen expression in primary activated PSC.

Unlike other human PSC lines that were immortalized from inflammation or non-neoplastic or neoplastic pancreatic specimens, HP-1 cell was established from normal pancreatic tissue obtained from a pancreatic cyst resection; thus, the primary PSCs were isolated by a multi-enzyme digestion strategy 6. HP-1 cells retained expression of proteins that are common in activated PSC, including α-SMA, vimentin, desmin and GFAP. GFAP has been proposed as a novel marker of PSCs which allows them to be distinguished from pancreatic fibroblasts 2, 3. HP-1 cells are stable after subculturing for at least 50 generations. Compared with other human PSC lines the advantages of HP-1 cells are stable growth in DMEM with 2.5% FBS at 37ºC and normal viability under serum-free condition within 48 h and high transfectability 8, 9.

TGF-β1, the major fibrogenic growth factor, is implicated in the biosynthesis of extracellular matrices and the formation of pancreatic fibrosis by activating PSCs 11-13. Recently, our studies indicate that TGF-β1 produced in an autocrine/ paracrine manner promotes rat PSC function, including cell migration, production of Col1, MMP-1 and MMP-2, and inhibition of TIMP-2 5, 13. Upon TGF-β1 stimulation, TGF-β type II receptor (TGFβR2) kinase phosphorylates TGFβR1 whereupon Smad2 and Smad3 phosphorylated 14. Studies have shown that TGF-β1 promotes PSC activation in a Smad2-dependent fashion, while TGF-β1/Smad3 pathway transmits signals to induce collagen synthesis and PSC activation 14, 15. BAMBI, a TGF-β family type I receptor, lacks an intracellular kinase domain and has the ability to block signal transduction after stimulation with TGF-β 13. Recently, we showed that LPS could enhance TGF-β1 signaling in rat PSC by repressing BAMBI via TLR4/MyD88/NF-kB activation 13. PDGF is a disulfide-linked dimer consisting of two peptides-chain A and chain B. It has three subforms: PDGF-AA, PDGF-BB, PDGF-AB. PDGF-BB which is consistent with predominant expression of the PDGF receptor β subunit has been found to be the most potent in stimulating PSC proliferation and migration, 6, 16, 17. PPARγ is a nuclear receptor that dimerises with retinoid-X-receptor to bind to DNA of target genes. Overexpression of PPARγ in PSCs inhibits proliferation and reduces collagen synthesis 7. In this study our result showed that HP-1 cells were highly consistent with PSCs in the expression of TGF-βRII, BAMBI, PDGFRβ and PPRPγ proteins and the regulation of matrix and matrix remodeling protein synthesis in response to TGF-β1.

Comparative proteomic analysis is a valuable method for an overall understanding of two or more cell protein profiles 2, 18. In 2013, Pauli et al compared the proteome of human hepatic stellate cells (hHSC) and human PSC using mass spectrometry (MS)-based quantitative proteomics. Among 1223 proteins, 1222 were found to be commonly expressed in both cell lines and a single protein was only detected in hHSCs. Of the 1222 quantified proteins, 888 were not significantly different in abundance between two cell types, 177 proteins were of higher abundance in hHSCs, while 157 were of higher abundance in PSCs. The authors suggested that use of enhanced MS instrumentation would allow greater proteome coverage, achieving a comparehensive proteomic analysis of hHSC and PSC 19. In 2015, Paulo et al reported 8100 quantitative proteins across nine human PSC samples using EASY-nLC 1000 LC-MS/MS (Orbitrap fusion) strategies 20. In this study, we determined protein profiles in six PSC or HP-1 cell samples using high-efficiency EASY-nLC 1000 LC-MS/MS (Q Exactive^TM^ Plus Orbitrap fusion). Our results show that there are 4,537 quantitative shared proteins between HP-1 cell and PSC. Proteins expressed solely by HP-1 cells or PSCs were not detected. Statistical analysis reveals no significantly different in matrices and matrix remodeling proteins. Only 9 proteins were up-regulated more than 2-fold in HP-1 cell and 13 proteins were up-regulated more than 2-fold in PSC, and these proteins did not include ECM proteins, cytokines, growth factors and matrix remodeling regulatory proteins.

In conclusion, we established an immortalized human PSC line HP-1 cell by using RSV promoter/enhancer-driven SV40 T antigen expression. HP-1 cells have characteristic cytoskeletal protein markers of PSC and demonstrate high transfeciency. HP-1 cells have characteristics consistent with activated PSC with respect to their expression of key receptors, regulation of matrices and matrix remodeling protein production in response to TGF-β1, and protein expression profiles. Thus we propose that HP-1 cells are an effective tool for the study of PSC-mediated pancreatic fibrosis.

## Supplementary Material

Supplementary materials.

## Figures and Tables

**Table 1:** Differentially expressed protein summary (Filtered with threshold value of expression fold change and P value < 0.05)

Compare group	Regulated type	1.5-2.0	2.0-3.0	3.0-4.0
HP-1/PSC	up-regulation	45	7	2
PSC/HP-1	up-regulation	32	10	3
