# Supplementary material for: Biological and Proteomic Characteristics of an Immortalized Human Pancreatic Stellate Cell Line
Source: Int J Med Sci. 2020 Jan 1;17(1):137–44. doi: 10.7150/ijms.36337 (PMC6945563; doi:10.7150/ijms.36337)
Supplement: Supplementary file 1 — Supplementary materials. [file ijmsv17p0137s1.pdf]

Supplementary material

Experimental procedures for isolation of human PSCs

1. Normal pancreatic tissue samples adjacent to the pancreatic cyst were obtained via routine pancreatic cystectomy.
2. The pancreatic tissue samples were placed in Hanks′ balanced salt solution (HBSS) without Ca^{++} and Mg^{++} containing 0.5 mM EGTA.
3. Centrifuge at 450 g for 5 min at 10 °C, and remove supernatant.
4. The tissue samples were placed in a Petri Dish and perfused with 100 ml DMEM without Ca^{++} and Mg^{++} including 0.03% collagenase P in an injection manner.
5. Centrifuge at 450 g for 5 min at 10 °C, and remove supernatant.
6. The tissues were pooled in 0.5 mM EGTA DMEM, minced with scissors.
7. Centrifuge at 450 g for 5 min at 10 °C, and remove supernatant.
8. The minced fragments of the tissue were digested with 10 ml DMEM containing 5 mg collagenase P and 2 mg hyaluronidase and protease ⅩⅣ for 20 min in a shaker at 37 °C (Dispersion by up-and-down suction through canulas with decreasing diameters).
9. Filter through 125 μm mesh
10. Filtrate was placed on top of 7.5 ml 0.3% BSA DMEM and centrifuged at 450 g for 5 min at 4 °C, removed supernatant.
11. Suspend the pellet in 8 ml 0.3% BSA DMEM.
12. The cell suspension was layered on the top of 21.36ml of 13.2% Optiprep/DMEM. Centrifuge at 1400 g for 10 min at 4 °C.
13. Collect a fuzzy band just above the interface and mix with 40 ml DMEM.
14. Centrifuge at 450 g for 7 min at 4 °C, remove supernatant.
15. The cells were resuspended in DMEM supplemented with 10% FBS, 25 mM HEPES buffer, and 100 U/ml penicillin/100 μg/ml streptomycin.
16. The cells were cultured at 37 ℃ in humidified atmosphere of 5% CO_2/95% air.
